# Changes in patient admission patterns at orthopedics and traumatology outpatient clinics before and after the earthquakes on 6 February 2023 in Turkey

**DOI:** 10.1186/s13018-023-03987-z

**Published:** 2023-07-11

**Authors:** Zeynel Mert Asfuroğlu, Ender Gümüşoğlu

**Affiliations:** Department of Orthopedics and Traumatology, School of Medicine, Mersin University, Çiftlikköy Campus, 33343 Yenişehir, Mersin, Turkey

**Keywords:** Earthquake, Orthopedics and traumatology, Outpatient clinic

## Abstract

**Background:**

The increase in orthopedic injuries after earthquakes imposes a significant burden on the health system. However, the impact of earthquakes on outpatient admissions remains unclear. This study compared patient admissions to the orthopedics and traumatology outpatient clinics before and after earthquakes.

**Methods:**

The study was conducted at a tertiary university hospital near the earthquake zone. In total, 8549 outpatient admissions were retrospectively analyzed. The study population was divided into pre-earthquake (pre-EQ) and post-earthquake (post-EQ) groups. Factors such as gender, age, city of origin, and diagnosis were compared between the groups. In addition, unnecessary outpatient utilization (UOU) was defined and analyzed.

**Results:**

The pre-EQ and post-EQ groups included 4318 and 4231 patients, respectively. The two groups had no significant differences in age and sex distribution. However, the proportion of non-local patients increased after the earthquake (9.6% vs. 24.4%, *p* < 0.001). UOU was the most common reason for admission in both groups. The distribution of diagnoses differed significantly between the pre-EQ and post-EQ groups, with an increase in the number of trauma-related diagnoses (15.2% vs. 27.3%, *p* < 0.001) and a decrease in UOU (42.2% vs. 31.1%, *p* < 0.001) after the earthquake.

**Conclusions:**

Patient admission patterns at orthopedics and traumatology outpatient clinics changed significantly after the earthquake. The number of non-local patients and trauma-related diagnoses increased, whereas the number of unnecessary outpatients decreased.

*Level of evidence* Observational study.

## Background

**Fig. 1:**
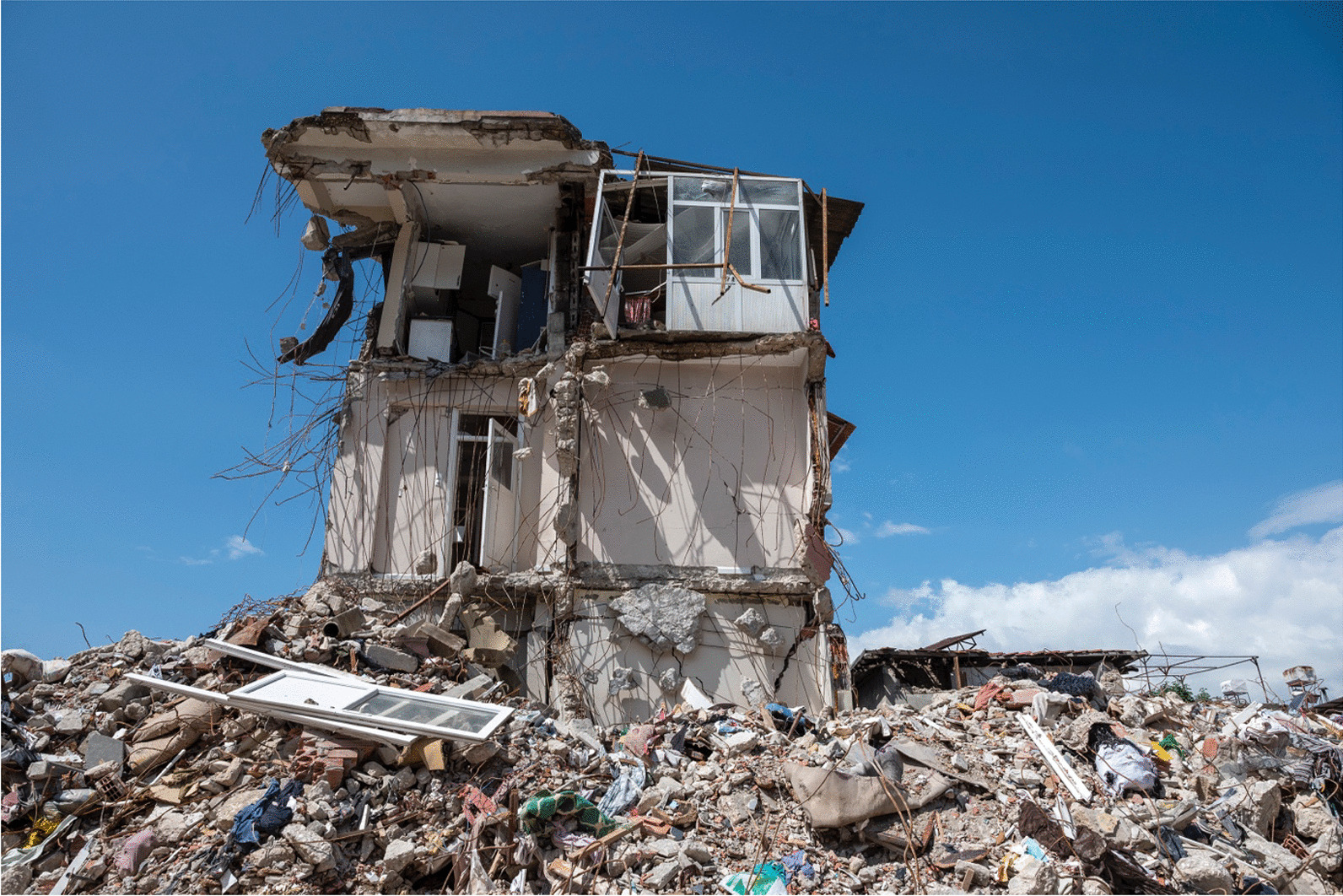
View from Hatay, one of the provinces most affected by the earthquake. (Used with permission from Murat Ünal)

Earthquakes are the most destructive form of natural disaster, with nearly 720,000 people worldwide losing their lives between 2000 and 2018 [1]. Turkey, located within the North Anatolian Fault Zone and the East Anatolian Fault Zone, has experienced numerous devastating earthquakes throughout its history, resulting in significant loss of life and property [2, 3]. On 6 February 2023, a 7.8-moment magnitude (Mw) earthquake struck southern and central Turkey. In Kahramanmaraş Province, nearly 9 h after the first earthquake, another earthquake with a magnitude of 7.6 Mw struck to the northeast of the initial epicenter. According to the Disaster and Emergency Management Presidency, the total death toll exceeded 50,000; accordingly, these earthquakes were called “the disaster of the century.” Moreover, this is the fifth deadliest earthquake of the twenty-first century [4]. (Fig. 1).

Earthquakes have a significant impact on healthcare systems [5]. Although a rapid increase in orthopedic injuries during the first few days is expected, the extensive damage to the surrounding area can have lasting health effects. Furthermore, the migration of people from the earthquake epicenter to nearby and safe provinces can lead to healthcare system shortages in unaffected areas.

The province of Mersin, located roughly 300 km away from the earthquake epicenter, remained undamaged, making it a new destination for the internal migration of earthquake victims. This migration has caused significant changes in the number of admissions to medical departments, particularly in orthopedics and traumatology, which are predominantly focused on treating earthquake-related injuries.

The impact of earthquakes on health care outcomes, such as outpatient admissions, has received limited attention in previous studies [6, 7]. Understanding the patterns of patient admissions following earthquakes is essential to enhance hospital preparedness and surge capacity for future disasters, and to facilitate a prompt return to daily routines.

This study compared changes outpatient admissions to the orthopedics and traumatology department between pre-earthquake (pre-EQ) and post-earthquake (post-EQ) groups.

## Methods

The Institutional Clinical Research Review Board of Mersin University approved the study protocol (2023/239). The study was conducted at a tertiary university hospital located near the earthquake zone; admissions to the orthopedics and traumatology outpatient clinics were retrospectively analyzed. Data were extracted from our hospital digital archive system. Diagnoses were recorded based on the International Classification of Diseases, 10th revision (ICD-10). The study included patients who visited the orthopedics and traumatology outpatient clinic and had their diagnostic codes entered into the digital archive system. Only the first admission was considered in the analysis of patients with multiple admissions at different times. The period 6–16 February 2023 was excluded, as no routine outpatient service was provided during this time. Two authors (ZMA and EG) recorded and classified the diagnosis.

### Study groups and parameters

This study compared patient admissions between the pre-EQ (6 December 2022–6 February 2023) and post-EQ (16 February 2023–16 April 2023) groups. The parameters analyzed included gender, age, city of origin, and diagnosis. Patients were categorized as local (coming from the same city as the hospital) or non-local (coming from other cities); cities of origin of non-local patients were analyzed. Diagnoses were classified into nine categories based on orthopedics and traumatology subspecialties (“trauma”, “spine”, “shoulder & elbow”, “knee & sports”, “pediatrics”, “reconstructive”, “hand”, “foot & ankle”, “oncology”). Furthermore, the archived information of patients with nonspecific diagnosis codes, such as M79 (other soft tissue disorders, not elsewhere classified) and M25.5 (pain in the joint), was evaluated. Patients who sought outpatient care for their needs, which could have been solved in another medical setting, such as primary care or preventive care, were classified as “unnecessary outpatient utilization (UOU)”. The patients classified as UOU did not have any specific orthopedic diagnosis.

### Statistical analyses

Descriptive data are presented as percentages, frequencies, means, and standard deviations. Categorical variables were compared using cross-tables and chi-square tests. The t-test was used to compare independent groups. A *p*-value < 0.05 was considered statistically significant.

## Results

In total, 8549 patients, including 4252 females and 4297 males, were included in the study. Of these, 4318 (2090 females and 2228 males) and 4231 (2162 females and 2069 males) patients were in the pre- and post-EQ groups, respectively. The mean age of the patients in the pre- and post-EQ groups was 38.3 and 40.4 years, respectively (Table 1). The proportion of non-local patients significantly increased in the post-EQ group (Table 1). The most frequent reason for admission in both groups was UOU (Table 1). The most common diagnoses were related to trauma and hand surgery in the pre- and post-EQ groups (Table 1). However, the distribution of diagnoses differed significantly between the two groups. In the post-EQ group, the number of trauma-related diagnoses increased, whereas the number of UOUs decreased significantly (Table 1). The city of origin distribution differed significantly between the pre- and post-EQ groups. Notably, the highest number of admissions was from Hatay Province (Table 2).

**Table 1:** Demographic and diagnostic characteristics of the patients in the pre-earthquake (pre-EQ) and post-earthquake (post-EQ) groups

	Pre-EQ group (n = 4318)	Post-EQ group (n = 4231)	Total (N = 8549)	*p* value
Age (mean ± SD)	38.3 ± 22.1	40.4 ± 21.9	39.8 ± 22.0	0.022^a^
*Gender, n (%)*				
Female	2090 (48.4)	2162 (51.1)	4252 (49.7)	0.007^b^
Male	2228 (51.6)	2069 (48.9)	4297 (50.3)	
*City of origin, n (%)*				
Local	3906 (90.4)	3201 (75.6)	7107(83.1)	< 0.001^b^
Non-local	412 (9.6)	1029 (24.4)	1441(16.9)	
*Diagnosis, n (%)*				
Trauma	657 (15.2)	1154 (27.3)	1811 (21.2)	< 0.001^b^
Spine	68 (1.6)	62 (1.5)	130 (1.5)	
Sholuder & elbow	170 (3.9)	144 (3.4)	314 (3.7)	
Knee & sports	168 (3.9)	209 (4.9)	377 (4.4)	
Pediatrics	196 (4.5)	182 (4.3)	378 (4.4)	
Reconstructive	204 (4.7)	221 (5.2)	425 (5.0)	
Hand	599 (13.9)	570 (13.5)	1169 (13.7)	
Foot & ankle	198 (4.6)	162 (3.8)	360 (4.2)	
Oncology	150 (3.5)	143 (3.4)	293 (3.4)	
Other	86 (2)	68 (1.6)	154 (1.8)	
Unnecessary outpatient utilization, n (%)	1822 (42.2)	1316 (31.1)	3138 (36.7)	< 0.001^b^

^a^Paired t-test, ^b^Cross-tables and chi-square tests. *p* < 0.05 was considered statistically significant

**Table 2:** Distribution of patient cities of origin

City of origin	Pre-EQ group (n = 4318)	Post-EQ group (n = 4231)	Total (n = 8549)	*p* value
Outside of the earthquake zone n (%)	4149 (96.1)	3343 (79.1)	7492 (87.6)	< 0.001
Earthquake zone n (%)	169 (3.9)	888 (20.1)	1057 (12.4)	< 0.001
Hatay	26 (0.6)	678 (16.1)	704 (8.2)	< 0.001
Adana	59 (1.4)	50 (1.2)	109 (1.3)	
Kahramanmaraş	13 (0.3)	35 (0.8)	48 (0.6)	
Gaziantep	16 (0.3)	27 (0.6)	43 (0.5)	
Osmaniye	13 (0.3)	10 (0.2)	23 (0.3)	
Malatya	6 (0.2)	23 (0.5)	29 (0.5)	
Diyarbakır	10 (0.2)	6 (0.1)	16 (0.6)	
Şanlıurfa	20 (0.4)	9 (0.2)	29 (0.3)	
Adıyaman	4 (0.1)	49 (1.2)	53 (0.6)	
Kilis	2 (0.1)	1 (0.0)	3 (0.0)	

Cross-tables and chi-square tests were used. *p* < 0.05 was considered statistically significant

## Discussion

In the present study, we assessed demographic and diagnostic-based changes in the orthopedic outpatient department during the pre- and post-EQ period. The total number of admissions remained unchanged, the rates of non-local patients and trauma-related diagnoses increased, and the rate of unnecessary admissions decreased after the earthquake.

Geography, demographics, and environmental conditions can influence patient admission patterns in orthopedic and traumatology outpatient clinics [8]. Natural disasters, such as earthquakes, can cause significant damage to medical infrastructure, loss of life, and demographic and environmental changes [9]. As a result, affected individuals often seek treatment at nearby hospitals that are in satisfactory operating condition [10]. These factors can cause changes in patient admissions at affected and unaffected hospitals. Health facilities should evaluate the changes in prevalence of diagnoses during the post-EQ period to provide efficient patient care. Although there are limited studies on changes in overall hospital admissions after earthquakes, no study has focused solely on orthopedic outpatient admissions. Moitinho de Almeida et al. [11] reported decreased hospital admissions during the post-EQ period. Ochi et al. [12] reported that the risk of healthcare clinic closures was significantly correlated with building damage. Although the hospital in our study was near the earthquake zone, no building damage was detected. Therefore, there was no significant change in the number of patient admissions during the post-EQ period at our facility.

Orthopedic and traumatology diagnoses can affect patients of all ages and sex, leading to variations in age and sex distribution among studies [13, 14]. In our study, the mean age of the patients in the pre- and post-EQ groups was 38.3 and 40.4 years, respectively, with an approximately equal sex distribution. These characteristics are consistent with the general population of Turkey [15].

The 6 February 2023 earthquakes resulted in significant population shifts in critical locations across the affected regions. Malatya (58%) and Hatay (60%) experienced the largest population declines, leading to a significant increase in population in provinces less affected by the earthquake [16]. Our study demonstrated the impact of this circumstance on the daily functioning of the orthopedic outpatient clinic. The rate of non-local patients in the Pre-EQ group was 9.6%, which increased to 24.4% in the post-EQ group (*p* < 0.005). (Table 1). Hatay province had the highest number of admissions (Table 2), indicating significant immigration from this province. There were two significant reasons for this migration. The first is that Hatay Province suffered the most from the earthquake, and the second is that people in Hatay Province prefer Mersin as a province in which to establish their new lives because of the sociocultural similarities. As a result of this internal migration, the local patient rate has decreased, indicating that it is becoming more challenging for local patients to access healthcare providers. Increasing the number of health service providers in regions outside of the earthquake zone would ensure that local patients receive continuous care after the earthquake.

Our orthopedic department is a leading referral center in the eastern Mediterranean region of Turkey, where four separate outpatient clinics receive approximately 200 patients daily. However, many of these admissions do not require primary care from an orthopedic specialist. Although not the primary objective of this study, we also investigated admissions not primarily related to orthopedics, referred to as “unnecessary outpatient utilization.” Our findings revealed a UOU rate of 36.7% (Table 1), which is higher than the rate reported by Azfar et al. [9] of 29.6%. Unfortunately, this issue with the Turkish health system has remained unresolved for many years.

The distribution of diagnoses among patients attending orthopedic outpatient clinics can vary. For instance, a pediatric orthopedics center would see admissions related to that field. In one of the largest trauma centers in India, Banerjee et al. [14] reported that trauma-related diagnoses were common. Similarly, Kumar et al. [17] found that trauma was a significant reason for admission for those aged 18–30 years, but low back pain was the most common diagnosis overall. In our study, the increased incidence of trauma and hand surgery diagnoses in both groups can be attributed to our clinic being a referral center for these specialties (Table 1).

Von Schreeb et al. [18] demonstrated the need for hospital care to address trauma-related complications after an earthquake. Our study revealed a twofold increase in trauma-related admissions after the earthquake, which significantly decreased UOU (Table 1). During the triage of outpatient clinic appointments, some patients with trauma-related injuries, as well as pediatric and older patients, were prioritized. Unnecessary admissions did not receive any appointments because the rate of trauma-related injuries increased significantly during the post-EQ period. Therefore, we hypothesize that the decrease in unnecessary admissions may be attributed to increased trauma-related admissions.

Our study had some limitations. The most significant limitation was the use of ICD-10 codes for diagnosing patients, which may be inadequate in some cases. We conducted detailed analyses in cases where the diagnosis code was ambiguous, and some diagnoses were classified as “other.” This approach maintained the integrity of the sample and reflected reality. Another limitation is that the study compared different periods within the same year because of missing information in our outpatient diagnostic records over the past few years. A more accurate comparison could have been made using the same period of different years. In addition, no studies in the literature have examined the functioning of orthopedic and traumatology outpatient clinics during an earthquake, making our study unique.

## Conclusion

In conclusion, the total number of orthopedic outpatient admissions did not change during the post-earthquake period. However, the rate of non-local patients increased, and the rate of local patients decreased. In the diagnosis-based analysis, the rate of trauma-related diagnoses increased the most. The rate of unnecessary admissions decreased during the post-earthquake period. We recommend that health systems near earthquake zones prepare accordingly for significant changes in patient populations after the earthquake.

## Data Availability

The datasets used and/or analysed during the current study are available from the corresponding author on reasonable request.

## Abbreviations

Mw: Moment magnitude
Post-EQ: Post-earthquake
Pre-EQ: Pre-earthquake
ICD-10: International classification of diseases, 10th revision
UOU: Unnecessary outpatient utilization
